# OD17 Future Health And Economic Burden Of Cardiovascular Disease In Type 2 Diabetes In Australia From 2021 To 2031

**DOI:** 10.1017/S026646232400151X

**Published:** 2025-01-07

**Authors:** Dina Abushanab, Clara Marquina, Jedidiah I Morton, Daoud Al-Badriyeh, Melanie Lloyd, Dianna J Magliano, Danny Liew, Zanfina Ademi

## Abstract

**Introduction:**

Diabetes is a key risk factor for cardiovascular disease (CVD), with a two-to-four-times higher risk than that in non-diabetes. The health burden associated with CVD imposes a major economic concern on healthcare systems and on society. We sought to estimate the future burden of CVD in terms of health and economic outcomes in type 2 diabetes (T2D) from 2022 to 2031.

**Methods:**

From Australian public healthcare and societal perspectives over the next decade, two dynamic models with annual cycles were designed to predict myocardial infarction (MI) and stroke in patients aged 40 to 90 years. First events at risk of CVD people were estimated using the 2013 pooled cohort equation, while recurrent events in existing CVD were acquired from the global Reduction of Atherothrombosis for Continued Health registry. Costs and utilities were obtained using public sources. Outcomes were fatal and non-fatal MI and stroke, years of life lived, quality-adjusted life years (QALYs), total direct and indirect costs. An annual discount rate of 5 percent was used.

**Results:**

Over the next 10 years, the model projected a total of 83,618 non-fatal MIs (95% confidence interval [CI]: 83,170, 84,053) and 58,774 non-fatal strokes (95% CI: 58,458, 59,013). In terms of health outcomes, total years of life lived and QALYs were 9,549,487 (95% CI: 9,416,423, 9,654,043) and 6,632,897 (95% CI: 5,065,606, 7,591,679), respectively. In terms of economic outcomes, total direct and indirect were AUD9.59 billion (USD6.38 billion) (95% CI: AUD1.90 billion [USD1.26 billion], AUD30.45 billion [USD20.26 billion]) and AUD9.07 billion (USD6.03 billion) (95% CI: AUD663.53 million [USD441.61 million], AUD33.19 billion [USD21.96 billion]), respectively. The chronic costs of CVD contributed most of the total direct cost, while morbidity contributed most of the total indirect costs.

**Conclusions:**

Our study shows that from 2022 to 2031, CVD as a complication from T2D will cause a significant impact on the Australian healthcare system and society. These estimates can be used to explore different strategies to optimize the control of risk factors for the prevention and management of CVD in T2D in Australia.

